# A Study Protocol on Risk Prediction Modelling of Mortality and In-Hospital Major Bleeding Following Percutaneous Coronary Intervention in an Australian Population: Machine Learning Approach

**DOI:** 10.3390/mps8060148

**Published:** 2025-12-05

**Authors:** Mohammad Rocky Khan Chowdhury, Mamunur Rashid, Dion Stub, Diem Dinh, Md Nazmul Karim, Baki Billah

**Affiliations:** 1 Department of Epidemiology and Preventive Medicine, School of Public Health and Preventive Medicine, Monash University, Melbourne, VIC 3004, Australia; mohammad.chowdhury2@monash.edu (M.R.K.C.); dion.stub@monash.edu (D.S.); diem.dinh@monash.edu (D.D.); nazmul.karim@monash.edu (M.N.K.); baki.billah@monash.edu (B.B.); 2 Department of Population Science, Jatiya Kabi Kazi Nazrul Islam University, Mymensingh 2224, Bangladesh; 3 Unit of Public Health Sciences, Faculty of Health and Working Life, University of Gävle, SE-80176 Gävle, Sweden; 4 Department of Cardiology, The Alfred Hospital, Melbourne, VIC 3004, Australia

**Keywords:** mortality, bleeding, prediction model, PCI, machine learning

## Abstract

Machine learning (ML) excels over regression by automatically capturing complex, non-linear relationships and interactions, enabling more flexible and accurate predictions without strict assumptions. This study focuses on developing ML-based predictive models for key post-PCI outcomes: 30-day mortality, in-hospital major bleeding, and one-year mortality. Data from 104,665 consecutive PCI cases in the Victorian Cardiac Outcomes Registry (VCOR), collected between 2013 and 2022, will be analyzed. Candidate variables, informed by prior systematic reviews and dataset availability, will undergo multiple imputations for missing values. The Boruta method will be applied to identify influential predictors. Risk-adjusted models will be developed using sophisticated ML algorithms, with performance compared across standard metrics for validation. The dataset will be split, optimized via 10-fold cross-validation, and class imbalance addressed using Adaptive Synthetic resampling technique. SHapley Additive exPlanations will interpret the most influential predictors. The variables from the best model will be converted into simplified numeric scores. External validation will be performed using the Tasmanian dataset or equivalent datasets. This study is expected to identify the most influential variables associated with 30-day all-cause mortality, in-hospital major bleeding, and long-term mortality post-PCI. These variables will form the basis for developing robust risk-scoring models to support clinical decision-making and outcome prediction.

## 1. Introduction

Coronary artery disease (CAD) remains a leading cause of mortality, responsible for over nine million deaths worldwide in 2016 [1]. In Australia, 0.6 million adults (2.8%) were living with CAD in 2018 [2], contributing to 78,000 years of potential life lost in 2019 [3]. On average, 440 Australians are hospitalized with CAD daily, about one every three minutes [4], accounting for 11% of the national disease burden [5].

Percutaneous coronary intervention (PCI) is the most common invasive cardiac procedure for treating obstructive CAD, including unstable angina, acute myocardial infarction (MI), and multivessel CAD [6]. Every year, around 45,000 PCIs are performed in Australia [7]. Although PCI is relatively safe as compared to other cardiac procedures such as bypass grafting, it is associated with some major post-PCI outcomes such as major bleeding, stroke, cardiogenic shock, recurrent MI or stent thrombosis, and target vessel revascularization and death [8,9].

To minimize procedural risks, greater emphasis is being given to the rational use of PCI by supporting clinical decision-making with risk-adjusted statistical modelling. Quantifying risk through scoring systems enables physicians to select the most appropriate intervention by balancing potential benefits against risks, tailored to each patient’s demographics, lifestyle, comorbidities, and clinical profile. This approach not only enhances treatment quality through improved risk adjustment but also promotes cost-effective care, thereby reducing post-PCI healthcare costs through optimized therapies and medication use [10,11].

Several risk assessment models for evaluating post-PCI adverse outcomes have been developed across diverse patient populations worldwide [12,13,14]. While these models are valuable in guiding clinical decisions regarding treating PCI, the underlying risk factors and their assigned weights vary considerably across algorithms due to differences in populations, treatment settings, and regional contexts [12,13,14,15,16]. Additionally, most existing risk scores, including those developed for the Australian population, have traditionally relied on multivariable Logistic Regression (LR) or Cox Proportional Hazards (CoxPH) models to predict post-PCI outcomes [12,13,14,15,16,17,18,19].

In recent years, there has been a growing interest in the use of machine learning (ML), a branch of Artificial Intelligence, to develop risk prediction models [20,21,22]. The ML algorithm efficiently deals with high-level non-structured big data from patient databases and is often able to detect sophisticated data patterns in a large dataset with a multitude of variables that can be tested for numerous interactions and non-linear relationships with outcomes that traditional statistical methods are sometimes unable to explain [10,23,24]. More explicitly, ML offers several advantages over traditional regression approaches for predicting post-PCI outcomes. First, many clinical and procedural variables, such as age or blood pressure, exhibit non-linear risk patterns and threshold effects that ML can model naturally without requiring manual transformations. Further, ML algorithms can automatically identify and learn complex high-order interactions among comorbidities (e.g., diabetes and renal impairment), procedural characteristics (e.g., lesion location), and presentation features, which would be impractical to pre-specify in regression models. Finally, ML methods are more robust when dealing with high-dimensional data (e.g., patients with multiple lesions) and correlated (e.g., systolic and diastolic blood pressure) predictors, whereas regression models may be affected by multicollinearity and instability.

ML algorithms have been widely applied to predict patient outcomes across various public health domains, including hospital readmission risk, cancer progression, and diabetic complications, and consistently demonstrate superior discrimination and calibration, even when variable relationships are complex [24,25,26]. However, using ML algorithms to predict post-PCI adverse outcomes is relatively new in the literature and has been far less studied and discussed in the context of the Australian PCI patient population. Therefore, this current study will address the following aims:

### 1.1. Overall Aim

The overall aim of the present study is to develop preprocedural risk prediction models using ML algorithms for post-PCI adverse outcomes, which include 30-day all-cause mortality, in-hospital major bleeding, and long-term survival/mortality.

### 1.2. Specific Aims

1. To develop an ML-based preprocedural risk prediction model for 30-day all-cause mortality post-PCI in an Australian population.
2. To develop an ML-based preprocedural risk prediction model for in-hospital major bleeding post-PCI in an Australian Population.
3. To develop an ML-based preprocedural risk prediction model for one-year mortality post-PCI in an Australian population.

## 2. Methods

### 2.1. Study Design and Population

The Victorian Cardiac Outcomes Registry (VCOR) prospectively collects data on all adults (≥18 years) undergoing PCI in Victoria, Australia. Established on 1 January 2013, the registry includes contributions from all 33 hospitals in the state, 15 public and 18 private, that perform PCI. For this study, data covering the period from 1 January 2013 to 31 December 2022, comprising 104,665 PCI procedures, will be analyzed [27].

### 2.2. The Victorian Cardiac Outcomes Registry Database

The Victorian Cardiac Outcomes Registry (VCOR) is a clinical quality registry monitoring outcomes and complications within 30 days post-PCI. It operates under Australian Monash University’s independent academic management in collaboration with key stakeholders and is guided by a Steering Committee with dedicated subcommittees [28]. Endorsed by the Australian Health Ministers’ Advisory Council in 2014, VCOR has received funding support from Monash University, Medibank Private, the Victorian Department of Health and Human Services, and the National Cardiac Registry Program [28].

The VCOR aims to facilitate the following [28]:

- A dataset aligned with national reporting guidelines,
- Developing a centralized mechanism for providing feedback,
- Benchmarking clinical outcomes and improving patient care,
- Areas of excellence and opportunities for improvement,
- Adequacy of access to resources available,
- Development of procedures, clinical guidelines, and policies,
- Insight into the safety of cardiac services,
- Identification of population groups that may require better access to care.

### 2.3. Overview of Model Development

This study will be conducted in accordance with the Transparent Reporting of a multivariable prediction model for Individual Prognosis Or Diagnosis (TRIPOD) guidelines [29]. In general, risk prediction models use predictors (variables) to estimate the absolute probability of an outcome or to relate an outcome of interest to a defined set of predictors [30]. These predictors may include demographic characteristics, physiological status, clinical presentation, comorbidities, and pre-procedural information. In the context of PCI, such models aim to estimate the risk of post-procedural mortality and morbidity prior to the intervention. In this current study, postprocedural 30-day all-cause mortality, in-hospital major bleeding, and long-term survival will be modelled using VCOR data. Strategies for model development include variable selection, treatment of missing data, development of a risk prediction model and its validation, and risk scoring estimation.

### 2.4. Variable Selection

For the primary purpose of this study, the candidate variables will be selected by conducting a systematic review of risk-adjusted models related to 30-day mortality and in-hospital mortality post-PCI and will be based on their availability in the VCOR dataset. Over the years, VCOR has consistently maintained completeness and coding consistency across hospitals. The operational definition of some potential variables in VCOR has been presented in Table 1, while the outcome variables and some potential independent variables are presented below.

### 2.5. Outcome Variables

The outcome variables in this study are dichotomous variables indicating whether a patient dies within 30 days, survives one year, or has an in-hospital major bleeding history after the PCI procedure. A 30-day mortality is a patient who dies within 30 days of the PCI. Any death within 12 months will be considered one-year survival or mortality and will be extracted from the National Death Index (NDI) [27]. This study will consider the definition of in-hospital major bleeding based on the Bleeding Academic Research Consortium (BARC) classification, which consists of five types of bleeding. Type 0 for no bleed; types 1 and 2 are minor bleed, and types 3 to 5 are clinically significant or major bleed [31].

Type 3a: Overt bleeding plus a hemoglobin drop of 3 to 5 g/dL (provided the hemoglobin drop is related to bleed); any transfusion with overt bleeding.

Type 3b: Overt bleeding plus a hemoglobin drop of 5 g/dL (provided the hemoglobin drop is related to bleed); cardiac tamponade; bleeding requiring surgical intervention for control (excluding dental, nasal, skin, and hemorrhoid); bleeding requiring intravenous vasoactive agents.

Type 3c: Intracranial hemorrhage (does not include microbleeds or hemorrhagic transformation, does include intraspinal); subcategories confirmed by autopsy, imaging, or lumbar puncture; intraocular bleed compromising vision.

Type 4: Coronary artery bypass grafting-related bleeding; perioperative intracranial bleeding within 48 h; reoperation after closure of sternotomy for the purpose of controlling bleeding; transfusion of 5 U of whole blood or packed red blood cells within a 48 h period; chest tube output 2 L within a 24 h period.

Type 5a: Probable fatal bleeding; no autopsy or imaging confirmation, but clinically suspicious.

Type 5b: Definite fatal bleeding; overt bleeding or autopsy, or imaging confirmation.

### 2.6. Exposure Variables

Demographics and anthropometric variables: Patient demographics, such as age and gender. The anthropometric measurement includes body mass index (BMI) (kg/m^2^).

Clinical variables: Clinical presentation aspect refers to information that potentially will be linked to physiological state or serious/life-limiting conditions, such as cardiogenic shock, out-of-hospital cardiac arrest (OCHA), or estimated Glomerular Filtration Rate (eGFR).

Comorbid variables: Comorbidities of interest will comprise factors that will be considered high-risk, such as history of diabetes, hypertension, Previous PCI, previous coronary artery bypass graft surgery (CABG), peripheral vascular disease, and cerebrovascular disease.

Pre-procedural medication variables: Information regarding pre-procedural medication includes glycoprotein IIb/IIIa (GP IIb/IIIa) inhibitor, or chronic oral anticoagulant therapy (COACT).

Procedural variable: Intraprocedural data will include percutaneous access site, lesion location, complex lesion, chronic total occlusion, and left ventricular ejection fraction (LVEF); they are determined from pre-procedural laboratory and imaging studies.

### 2.7. Treatment of Missing Data

The characteristics of missing and non-missing patients were compared and tested for whether missingness is completely at random (MCAR) using Little’s test, as well as for the presence of covariate-dependent missingness. Missing values occur when data for a particular variable are not recorded in an observation of interest. Addressing these gaps through imputation can enhance model performance [32]. When data are missing, the effective sample size decreases, which may produce biased or inaccurate parameter estimates and, ultimately, misleading conclusions [33]. If the proportion of missing data for any variable is below 20%, multiple imputation is an appropriate method for handling the missing values [34]. In this study, missing values will be handled using the Multiple Imputation by Chained Equations (MICE) method with fully conditional specification [35]. The chained equations approach can handle variables of various types and complexities [36].

Missing values are filled in by using the available data for a particular individual and considering relationships seen in the data for other participants, as long as the variables used for imputation are present. Multiple imputations generate several predictions for each missing value, enabling analyses to estimate the uncertainty in the imputations and provide accurate standard errors [37,38].

If missingness is systematic, bias may remain. To address this, a sensitivity analysis will be performed by comparing results from complete-case analysis with those obtained using imputation for each individual analysis.

### 2.8. Analyses

Patients’ characteristics will be summarized as mean ± standard deviation (SD) or, where appropriate, median with percentiles for numerical variables, and as percentages for categorical variables. Multicollinearity will be examined using the Variance Inflation Factor (VIF), with a threshold of VIF > 10 indicating multicollinearity [39]. Additionally, first-degree interaction effects between clinically relevant risk factors will be explored using (LR) analysis. The association between outcomes and independent variables will be evaluated using the Chi-square test or Fisher’s exact test, with statistical significance set at *p* < 0.05.

The Boruta variable selection method will be applied to identify the most relevant variables from the initial set of factors. Boruta is a wrapper-based approach built on the random forest algorithm, recognized for its consistency and unbiased performance, making it superior to many other feature selection techniques [40]. Using the selected variables, several ML models will be developed for each outcome. The most influential predictors will then be ranked and interpreted with SHapley Additive exPlanations (SHAP) [41]. SHAP is a widely used tool for interpreting ML models, as it attributes importance to individual factors and explains their contribution to predictions. While not a direct feature selection method, SHAP facilitates understanding of variable influence, guiding decisions on inclusion by ranking predictors, visualizing their impact, applying thresholds, and iteratively refining the set of variables.

**Figure 1:**
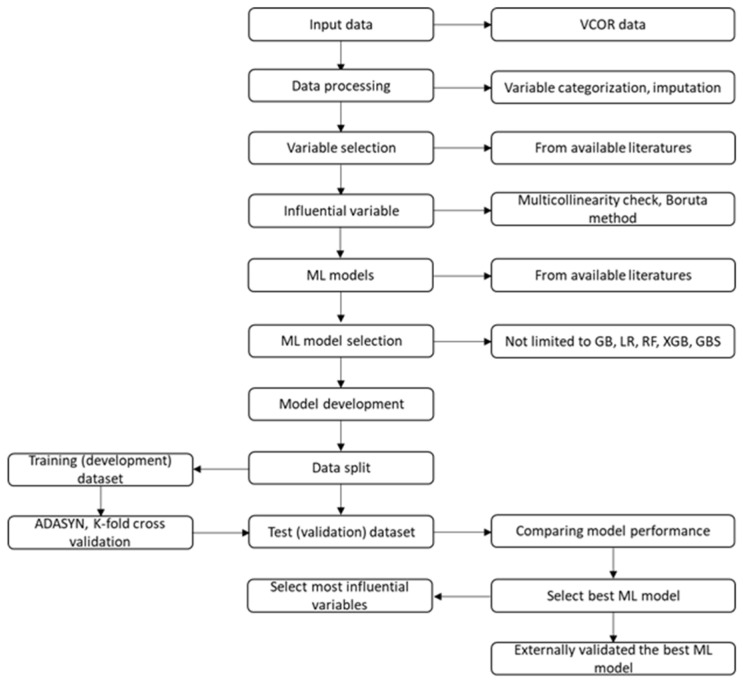
Schematic presentation of machine learning model development.

The potential risk factors selected by the best ML models will be entered into the regression models (traditional LR and CoxPH models) to quantify the risk of predictors based on a significance threshold of *p*-values of less than or equal to 0.05. A schematic presentation of the selection of the best ML model is shown in Figure 1.

### 2.9. Development of ML-Based Risk Prediction Models

Developing a risk prediction model typically involves selecting a set of influential predictors from predefined candidate variables, assigning relative weights to each predictor to construct a composite risk score, evaluating the model’s performance, and assessing potential optimism through internal validation techniques, with adjustments for overfitting applied as needed [30].

Initially, potential predictor variables will be identified through a review of the literature and consultation with an interventional cardiologist, focusing on the variables available in the VCOR dataset. Subsequently, only the preprocedural and intraprocedural patient characteristics deemed influential by the Boruta method will be included in the model development. For model development, the dataset will be randomly split into 70% for the training dataset and 30% for the test dataset. To address the class imbalance of the outcomes, the Adaptive Synthetic (ADASYN) resampling technique will be applied. ADASYN creates synthetic samples for the minority class, boosting its representation and making its size more comparable to that of the majority class. This results in a more balanced dataset, helping to mitigate the bias caused by the initial class imbalance and enhancing the model’s robustness. Moreover, each training model was optimized with hyperparameter tuning using a 10-fold cross-validation protocol. In this protocol, the trained dataset will be randomly divided into 10 equal parts for 10 iterations. The algorithm undergoes development (90% of data) and validation (10% of data) for each part in the trained dataset, and the performance metrics are aggregated over 10 iterations to yield an overall performance score. Subsequently, the optimized training models were validated in a 30% test dataset.

Additionally, the dataset will be split into two time-based subsets, 2013–2018 for training and 2019–2022 for testing, to address temporal variation, acknowledging that procedural techniques and medications have evolved over time. The best ML model was selected based on a comparison of their performance metrics (accuracy, root mean square error (RMSE), sensitivity/recall, specificity, precision, F1 score, Brier score/integrated Brier score, receiver operating characteristics—area under the curve (ROC-AUC) with 95% confidence interval (CI), PR score, and calibration plot) in a 30% test dataset (Table 2). The best-performing model will be selected based on its superiority across most evaluation metrics, including the ROC–AUC score.

To develop the models, this study will employ several ML algorithms that have been shown to be promising in the previous literature and which are appropriate for dichotomous non-time-to-event or time-to-event-outcomes [42,43,44,45,46]. For binary, non–time-to-event outcomes (e.g., 30-day mortality or in-hospital major bleeding), the analysis will primarily employ classifier models such as Gradient Boosting (GB), LR, Random Forest (RF), and Extreme Gradient Boosting (XGB), among others [42,44]. For binary time-to-event outcomes, ML–based CoxPH, Gradient Boosting Survival (GBS), Multilayer Perceptron (MLP), or Random Survival Forest (RSF) will be used [45,46,47,48,49,50]. The potential parameter optimization has been presented in Table 3.

CoxPH: A ML–based CoxPH model is an extension of the traditional Cox regression used for survival analysis, where the outcome is time-to-event data (e.g., time to death). The model estimates the hazard (risk) of an event occurring at a particular time, given a set of predictor variables, while assuming that the ratio of hazards between groups remains constant over time (the proportional hazards assumption). In the ML context, CoxPH can be enhanced with techniques such as regularization (Lasso, Ridge, Elastic Net) to handle high-dimensional datasets, prevent overfitting, and improve predictive accuracy. It can also be integrated into ML frameworks for model comparison, hyperparameter tuning, and cross-validation. Clinically, ML-based CoxPH is useful because it not only predicts whether an event will occur but also when it is likely to occur, making it highly relevant for guiding patient monitoring, treatment planning, and long-term outcome prediction.

GB: GB constructs an ensemble of decision trees to make accurate predictions. The process begins with an initial model and defines a loss function to measure prediction errors. Through iterative steps using gradient descent, new decision trees are added to the ensemble to correct previous errors, with each tree focusing on the residuals of the previous ones. Regularization techniques are applied to prevent overfitting, and the final prediction is made by aggregating the predictions of all trees. This mechanism allows gradient boosting to create powerful predictive models with strong generalization capabilities across various tasks, such as regression and classification.

GBS: GBS, also known as gradient boosting machines for survival analysis, are advanced techniques used in survival analysis to predict time-to-event outcomes. These methods employ gradient boosting algorithms, which iteratively combine weak learners (decision trees) to create a robust predictive model. The process involves minimizing a specific loss function, such as the negative log partial likelihood, through gradient descent. The algorithm adds new decision trees iteratively, focusing on residuals and leveraging regularization techniques to prevent overfitting. This model provides accurate predictions of survival probabilities or hazard rates while handling censored data effectively, making them valuable tools for analyzing time-dependent outcomes in various fields such as healthcare, finance, and engineering.

LR: A ML–based LR model is a statistical learning method used to predict binary outcomes (e.g., survival vs. death, bleeding vs. no bleeding) based on one or more predictor variables. While traditional LR estimates the probability of an outcome using a linear combination of predictors and provides coefficients with odds ratios, in the ML setting, LR is treated as a baseline classification algorithm within a broader predictive modelling framework. In ML pipelines, LR is often enhanced with regularization techniques (like L1/Lasso or L2/Ridge) to handle high-dimensional data, prevent overfitting, and improve generalizability. Though simpler than complex models such as Random Forest or Gradient Boosting, ML-based LR is valued for its interpretability, computational efficiency, and robustness, making it a reliable reference model when developing and comparing risk prediction tools.

MLP: An MLP is a class of feedforward artificial neural network consisting of an input layer, one or more hidden layers, and an output layer. Each layer is made up of interconnected neurons that apply non-linear activation functions, enabling the network to model complex, non-linear relationships in data. MLPs are fully connected, meaning each neuron in one layer is connected to every neuron in the next layer. They are trained using backpropagation and gradient descent to minimize a loss function. While MLPs are general-purpose models used for classification and regression, in survival analysis, they can be adapted to predict risk scores, survival probabilities, or hazard functions by replacing the output layer and loss function accordingly.

RF: Random Forest is an ensemble learning algorithm used for classification and regression tasks. It constructs multiple decision trees by bootstrap sampling from the dataset and randomly selecting features at each node. The algorithm aggregates predictions from these trees to make final predictions, improving accuracy and reducing overfitting. Random Forest’s randomness and diversity help capture complex relationships in the data, making it a popular choice for various ML applications.

RSF: Random Survival Forest is an extension of the Random Forest algorithm designed specifically for survival analysis, which deals with time-to-event data. The model constructs an ensemble of decision trees using bootstrap sampling and considers both time and event status at each node during tree construction. It predicts survival probabilities or hazard rates for each observation based on the ensemble of trees. Random Survival Forest handles censored data, provides insights into variable importance, and offers flexibility with tunable hyperparameters. It is a powerful and widely used algorithm for modelling time-dependent outcomes in various domains such as biomedical research, clinical studies, and reliability analysis.

XGB: Extreme Gradient Booster is a state-of-the-art algorithm used for supervised learning tasks such as regression, classification, and ranking. It employs a gradient boosting framework to sequentially build an ensemble of decision trees, optimizing a specified loss function. Key features of the model include optimized implementation for speed and efficiency, regularization techniques to prevent overfitting, parallel computing for faster computations, and innovative methods for split finding and tree pruning. The algorithm also handles missing values, provides insights into feature importance, and supports early stopping to improve model generalization. Overall, Extreme Gradient Booster is known for its superior performance, scalability, and ability to handle complex datasets, making it a popular choice in ML applications.

### 2.10. Presentation of the Model

Firstly, the final prediction model will be presented as the LR or CoxPH model equation. Finally, the estimated parameter values (beta coefficient) will be converted and rounded up to numbers as a simplified scoring rule to inform absolute outcome probabilities. The final prediction models can be made available as an online calculator or as a nomogram. The models’ interpretation will not conflict with the algorithmic reproducibility, as they will be transparently documented. The newly developed calculators or nomograms will be shared with clinicians and patient representatives to test their applicability in external clinical settings. Further, the study findings will be communicated in peer-reviewed journals and academic conferences to inform further research and policymaking about patients with PCI.

### 2.11. External Validation

The risk prediction models developed in this study for 30-day mortality, in-hospital major bleeding, and long-term mortality will undergo external validation using a Tasmanian dataset representing a different cohort. Additionally, future collaboration is planned with an Asian registry, such as the Malaysian National Cardiovascular Disease Database.

### 2.12. Healthcare Services’ Performance

The effectiveness of the best-performing models in predicting post-PCI outcomes will be evaluated by assessing the performance of health services using a funnel plot. Funnel plots will be employed to visually assess health service performance based on patients’ risk-adjusted probability of post-PCI outcomes, calculated using traditional LR. By plotting risk-adjusted rates of outcomes against the total number of procedures performed, these plots provide a useful tool for monitoring and benchmarking performance [51].

### 2.13. Statistical Software

Statistical software packages Stata (version 18), R (version 4.5.1), and Anaconda Distribution 2025.06-1 will be used for the analysis.

### 2.14. Strengths and Limitations of This Study

- This study will utilize registry data comprising a large cohort of patients who underwent percutaneous coronary intervention across 33 specialized hospitals in Victoria, Australia.
- A number of sophisticated ML algorithms will be employed to develop the risk prediction models.
- The risk prediction models will undergo further validation using external data.
- The study sample will consist solely of patients from Australia, which may introduce a cultural gap and restrict the generalizability of our findings.
- The study will rely on registry data, which may restrict the inclusion of certain variables previously identified as important.

## 3. Discussion

Using the VCOR dataset, a 30-day all-cause mortality post-PCI risk prediction model will be developed for an Australian population, with a recommendation for applying ML to update the model [18]. Previous traditional regression-based risk prediction models using VCOR data up to 2017 for the Australian population identified key variables for 30-day mortality risk adjustment, including cardiogenic shock, intubated OHCA, estimated GFR, LVEF, angina type, mechanical ventricular support, age ≥ 80 years, lesion complexity, percutaneous access site, and PVD [18]. Another regression-based model using the VCOR dataset among ACS patients with cardiogenic shock reported that age (per 10-year increase), female sex, diabetes, eGFR, OHCA, pre-procedural intubation, mechanical circulatory support, STEMI, and multivessel PCI were significant predictors of 30-day mortality [19]. To date, no ML-based risk prediction models are available for post-PCI outcomes, including 30-day mortality, one-year mortality, or in-hospital major bleeding, in the Australian population. This study proposes a methodology for developing risk prediction models to predict mortality and in-hospital major bleeding post-PCI. The models will be constructed using the same data from up to 2022 and will incorporate sophisticated ML algorithms to enhance predictive accuracy.

The potential predictors available within the dataset that will be considered for model development include age, gender, BMI, ACS, cardiogenic shock, OHCA, estimated GFR, LVEF, ACS, mechanical ventricular support, diabetes, GP IIb/IIIa inhibitor, and COACT. These factors are clinically important in assessing post-PCI adverse outcomes. Older age increases risk due to frailty and comorbidities [52], while female gender is associated with smaller vessels and higher bleeding susceptibility [53]. Higher BMI affects recovery and drug metabolism [54]. ACS presentation, cardiogenic shock, and OHCA indicate severe cardiac compromise and are strong predictors of mortality [55,56]. Renal impairment and reduced LVEF reflect poor organ and cardiac function, increasing both mortality and bleeding risks [57]. The need for mechanical ventricular support signals hemodynamic instability, further elevating risk [58]. Diabetes contributes to vascular and platelet dysfunction, predisposing patients to adverse outcomes [59]. Use of GP IIb/IIIa inhibitors improves ischemic control but heightens bleeding potential [60]. Further, complex lesions make procedures longer and more technically demanding, leading to greater procedural and bleeding risks [61].

Developing risk prediction models for mortality and major bleeding following PCI holds significant value, as the derived risk scores based on key predictive factors will enable individualized risk assessment, support clinical decision-making, enhance patient care, and promote the efficient use of healthcare resources. Particularly, these models allow clinicians to assess individual risk levels, guide pre-procedural planning, and tailor treatment strategies, potentially improving outcomes through early intervention. Hospitals can also use them to allocate resources more efficiently, prioritizing high-risk patients who may need closer monitoring or ICU admission. In addition, the models support effective communication with patients and families by providing realistic risk estimates, fostering shared decision-making. Beyond individual care, prediction models can inform updates to clinical guidelines, help evaluate institutional performance by comparing predicted versus actual outcomes, and promote continuous quality improvement across healthcare systems.

## Figures and Tables

**Table 1:** Description and discretization of variables.

Variables	Definition	Scale of Measurement	Type
**Demographics and anthropometric variables**			
Age (group in years)	The date of birth of the patients was collected in DD/MM/YYYY format.	<80 years and ≥80 years	Binary
Gender	The gender (sex) of the patients	Male, female	Binary
Body mass index (BMI)	Body mass index was calculated using the following formula: BMI = Weight in kg/(Height in meters)^2^ kg/m^2^. Weight was measured in kg in light clothing and height in centimetres in bare or stockinged feet. Height and weight measurements could be self-reported.	Underweight (<18.5 kg/m^2^), normal (18.5–24.9 kg/m^2^), Overweight (25.0–29.9 kg/m^2^), obesity (30.0 kg/m^2^ and above)	Categorical
**Clinical variables**			
Acute coronary syndrome (ACS)	ACS encompasses clinical features comprising chest pain or overwhelming shortness of breath, defined by accompanying clinical, ECG, and biochemical features. Specifically, ACS refers to unstable angina, non-ST-Elevation Myocardial Infarction (NSTEMI), and/or ST-Elevation Myocardial Infarction (STEMI). The patient must have experienced an ACS event within the last 7 days to be coded “yes” for ACS at the time of the PCI event.	No, yes	Binary
Cardiogenic shock	Cardiogenic shock is coded as ‘yes’ if all of the following apply: 1. Sustained (>30 min) episode of systolic blood pressure <90 mm Hg (or vasopressors required to maintain BP > 90 mm Hg); AND 2. Evidence of elevated filling pressures (e.g., pulmonary congestion on examination or chest radiograph); AND 3. Evidence of end-organ hypoperfusion (e.g., urine output 30 mL/h; or cold/diaphoretic extremities; or altered mental status, etc.).	No, yes	Binary
Out-of-hospital cardiac arrest	Cardiac arrest is coded as ‘yes’ if any one of the following applies: 1. If the patient has experienced an out-of-hospital cardiac arrest (i.e., the lack of effective cardiac output), including if the person was under cardiac arrest at the time of presentation to the hospital and/or the patient was intubated prior to the PCI procedure. and 2. If the patient was intubated prior to the PCI procedure.	No, yes	Binary
estimated Glomerular Filtration Rate (eGFR)	Record the last serum creatinine levels recorded within 60 days prior to the current PCI (in μmol/L). To convert from mmol/L to μmol/L, multiply by 1000 or move the decimal point 3 spaces to the right. The formula for eGFR uses age, gender, and the level of creatinine in blood to estimate GFR. eGFR of 90 or higher is in the normal range. eGFR of 60–89 may mean early-stage kidney disease/mild. eGFR of 30–59 may mean kidney disease/moderate. eGFR below 30 may mean kidney failure/severe	Normal, mild, moderate, severe	Categorical
Mechanical ventricular support	If a patient required mechanical ventricular support prior to the current PCI procedure.	No, yes	Binary
**Comorbid variables**			
Diabetes	If the patient had a current medical diagnosis of diabetes that required medical intervention (regardless of duration of the disease). This includes a medical diagnosis made during the current admission where medication is prescribed. The patient must be on anti-diabetic medication to lower blood sugar. Antidiabetic medications to lower blood sugar include oral hypoglycaemics and insulin. For patients whose diabetes is controlled with diet alone, code as ‘no’.	No, yes	Binary
Peripheral vascular disease (PVD)	If the patient displays evidence of either chronic or acute PVD. The presence of PVD must be demonstrated by vascular reconstruction or amputation for arterial insufficiency, bypass surgery, or percutaneous intervention. PVD includes the aorta, extremities, and carotid vessels.	No, yes	Binary
Cerebrovascular disease (CVD)	Indicate whether the patient has a history of stroke or cerebrovascular accident resulting from an ischaemic or intracerebral haemorrhagic event, only where the patient suffered a loss of neurological function with residual symptoms remaining for at least 72 h.	No, yes	Binary
Previous percutaneous coronary intervention (PCI)	If the patient has had a prior Percutaneous Transluminal Coronary Angioplasty, Coronary Atherectomy, and/or coronary stent performed at any time prior to the current PCI procedure.	No, yes	Binary
Previous coronary artery bypass grafting (CABG)	If the patient has had a prior CABG.	No, yes	Binary
**Pre-procedural medication variables**			
Glycoprotein IIb/IIIa inhibitor therapy	Agents include abciximab, eptifibatide, and tirofiban		
Aspirin	Agents include aspirin, astrix, cardiprin, cartia, assasantin, aspro, disprin, and solprin.		
**Procedural variable**			
Lesion location	Indicate the coronary segment that applies to each coronary lesion attempted during the current PCI. Every coronary lesion attempted during the current PCI must be recorded separately. Up to five lesions per PCI can be recorded. Right coronary artery Left anterior descending Circumflex artery Left main Graft	Right coronary artery, left anterior descending, circumflex artery, left main, graft	Categorical
Lesion complexity	Lesion type according to the ACC/AHA classification guideline for current lesion. Type A: Minimally complex, discrete (<10 mm), concentric, readily accessible, lesion in non-angulated segment (<45 degrees), smooth contour, little or no calcification, less than total occlusion, not ostial in location, no major side branch involvement, absence of thrombus. Type B: Only one type B characteristic—lesion moderately complex, tubular (10–20 mm), eccentric, moderately tortuosity of proximal segments, lesion in moderately angulated segment (>45 degrees but <90 degrees), irregular contour, moderately to heavy calcification, total occlusion less than three months old, ostial in location, bifurcation lesions requiring double guide wires, some thrombus present. Type B2: More than one type B characteristic Type C: severe complex diffuse (>20 mm), excessive tortuosity of proximal segment, lesion in extremely angulated segment >90 degrees, total occlusion greater than 3 months old or bridging collaterals, inability to protect major side branches, degenerated vein graft with friable lesion.	Type A and B Type B2 and C	Binary
Chronic total occlusion	Indicate whether the current lesion was presumed to be a chronic total occlusion. Chronic total occlusion is defined as being >3 months old and/or bridging collaterals.	No, yes	Binary
Left Ventricular Ejection Fraction (LVEF)	For all patients (excluding STEMIs), indicate whether the patient’s ventricular ejection fraction (EF) was measured (or estimated) within 6 months prior to the current procedure and up to four weeks post-discharge. This includes the period leading up to and including the cardiac catheter lab visit, after the lab visit, and up to four weeks after the patient was discharged. If multiple test results are available during this period, select the test result closest to the date/time of the current PCI. For STEMI patients, a LVEF test must have been recorded during the current admission or up to four weeks post-discharge for this item to be coded ‘yes’. For these patients, where no LVEF test was performed during the current admission or up to four weeks post-discharge, code “no”. EF tests include, but are not limited to, angiography, echocardiography, nuclear stress tests, and imaging scans.	No, yes	Binary

**Table 2:** Definition of model’s performance metrics.

True positive	A true positive is an outcome where the model correctly predicts the positive class
True negative	A true negative is an outcome where the model correctly predicts the negative class
False positive	A false positive is an outcome where the model incorrectly predicts the positive class
False negative	A false negative is an outcome where the model incorrectly predicts the negative class.
Accuracy	Accuracy tells us the overall proportion of correct predictions made by the model (both true positives and true negatives). Model accuracy is defined as the number of classifications a model correctly predicts divided by the total number of predictions made, which can be represented as: Accuracy = (True positives + True negatives)/(True positives + True negatives + False positives + False negatives). Higher accuracy indicates higher predictive performance
Sensitivity/recall	Sensitivity measures the model’s ability to correctly identify patients who truly have the outcome (e.g., mortality or major bleeding) Sensitivity refers to a test’s ability to designate an individual with a given disease as positive. A highly sensitive test means that there are few false negative results, and thus fewer cases of disease are missed. Sensitivity = true positive/(false negative + true positive) Higher sensitivity indicates higher predictive performance
Specificity	Specificity measures the model’s ability to correctly identify patients who do not have the outcome. Specificity measures the proportion of true negatives that are correctly identified by the model. Specificity = true negative/(true negative + false positive) Higher specificity indicates higher predictive performance
Precision	Precision measures the proportion of correctly predicted positive cases out of all predicted positive cases Precision ensures clinicians trust the model’s positive predictions, making it valuable for decision-making and resource allocation. High precision indicates that when the model flags a patient as “high risk” (or the test gives a positive result), it is usually correct, whereas low precision means that many patients identified as “at risk” do not actually experience the outcome, leading to false alarms and potentially unnecessary interventions. The formula for precision (also called positive predictive value, PPV) is: Precision = True positive/(True positive + False positive) A higher precision value indicates higher predictive performance
F1 score	The F1 score is the harmonic mean of precision and recall, balancing both metrics. It is calculated as follows: F1 = 2∗(Precision*recall)/(Precision + Recall) The F1 score provides a balanced measure of a model’s ability to correctly identify patients at risk while minimizing false alarms. It combines both precision (how often a predicted “high risk” is truly correct) and recall/sensitivity (how well the model captures all true high-risk patients). A high F1 score means the model not only identifies most patients who will develop the outcome but also avoids over-predicting risk, making it useful in guiding clinical decision-making where both missed cases and false positives carry important consequences. Interpretation: F1 = 1 → Perfect precision and recall (ideal model). F1 = 0 → The model has either zero precision or zero recall (worst performance). Higher F1 Score → Better model performance, meaning it effectively identifies positive cases while minimizing false positives and false negatives. Lower F1 Score → Poor performance, indicating the model struggles with either false positives or false negatives. The F1 score is crucial for imbalanced datasets, where accuracy alone can be misleading.
Receiver Operating Characteristics (ROC)	The ROC-AUC reflects how well a risk prediction model can discriminate between patients who will experience an adverse outcome (e.g., death or major bleeding) and those who will not. An AUC close to 1.0 indicates excellent discrimination, meaning the model reliably assigns higher risk scores to patients who truly experience the outcome compared to those who do not. An AUC around 0.5 suggests no better than chance, while values between 0.7 and 0.8 are considered acceptable and above 0.8 good for clinical use. In practice, a higher ROC-AUC means clinicians can more confidently use the model for risk stratification, tailoring interventions, and improving patient management decisions. The plot of sensitivity versus 1-Specificity is called the receiver operating characteristic (ROC) curve and the area under the curve (AUC). The mathematical formula of AUC is as follows: ROC *=* $\int_{x=0}^{1}[Sensitivity\;\{{\left(1-Specificity\right)}^{-1}\;\left(x\right)\}]dx$
Concordance Index (C-index)	The C-index is a commonly used metric to evaluate the predictive accuracy of survival models. It measures the model’s ability to correctly rank pairs of individuals based on their predicted risk scores relative to their actual survival times. Specifically, it estimates the probability that, for a randomly selected pair of patients, the one who experiences the event earlier also has a higher predicted risk. The C-index ranges from 0.5 (no better than random chance) to 1.0 (perfect prediction). It naturally handles censored data by only considering comparable pairs where the order of events can be determined. A higher C-index indicates a better discriminative ability of the survival model when distinguishing between high-risk and low-risk individuals. The C-index for survival data can be expressed as follows: $C\;=\;\frac{1}{N}\;\sum_{i,j}1\left(\hat{h_{i}}-\hat{h_{j}}\right).1\left(T_{i}<T_{j}\right).\partial_{i}$ where *Ti* and *Tj* are the observed survival times for individuals i and j, *δi* is the event indicator for individual i (1 if event observed, 0 if censored), are predicted risk scores (higher means higher risk), 1(⋅) is the indicator function, The summation is overall comparable pairs (i,j), meaning pairs where *Ti* < *Tj* and i is uncensored, N is the total number of comparable pairs.
Brier score	The Brier Score is a measure of the accuracy of probabilistic predictions. It evaluates how close the predicted probabilities are to the actual binary outcomes. Brier score = 1/N$\sum_{i=1}^{N}{\left(f_{i}-o_{i}\right)}^{2}$ where N = Total number of predictions f_i_ = Predicted probability for instance iii o_i_ = Actual outcome (1 for positive, 0 for negative) The Brier Score ranges from 0 to 1, where 0 indicates perfect predictions, 1 represents completely incorrect predictions, and lower scores signify better calibration and accuracy of probabilistic predictions. In practice, a lower Brier score indicates that clinicians can trust the model’s probability estimates for individual patients, making it more useful for guiding shared decision-making, personalized care, and risk communication with patients.
Integrated Brier Score (IBS)	The IBS is a metric used to evaluate the overall accuracy of survival models over time. It measures the mean squared difference between the predicted survival probabilities and the actual survival status, integrated over a specified time interval. The Brier score at a specific time t is calculated as follows: BS(t) = $\frac{1}{N}\;\sum_{i=1}^{N}w_{i}\left(t\right)\;(1\left(T_{i}>t\right)-\hat{S}(t|X_{i}){)}^{2}$ where N is the number of individuals, Ti is the observed survival time for individual i, $\hat{S}(t|X_{i})$ is the predicted survival probability at time t for individual i, 1(Ti > t) is an indicator that the event has not occurred by time t, $w_{i}(t)$ is a weighting function that accounts for censoring, often based on inverse probability of censoring weights. The IBS is the time-integral of the Brier score over the interval [0, τ], usually normalized by the length of the interval: IBS = $\frac{1}{\tau }\;\int_{0}^{\tau }BS\left(t\right)dt$ Lower IBS values indicate better prediction accuracy, with 0 being perfect prediction.

**Table 3:** Models’ parameter optimization.

Items	Hyperparameter Optimization	Cross-Validation (k-Fold)	Parameters	Thresholds
Gradian Booster (GB)	Grid search	10 folds	n_estimators, min_samples_split, min_sample_leaf, learning_rate, learning_rate, max_features, sub_smaple, scoring, n_jobs, random_state	0.5
Logistic Regression (LR)	Grid search	10 folds	random_state, C, penalty, solver, scoring	0.5
Random Forest (RF)	Grid search	10 folds	n_estimators, min_samples_split, max_features	0.5
Extreme Gradient Booster (XGB)	Grid search	10 folds	random_state, objective, alpha, gamme, eval_metrics, eta, learning_rate, max_delta_step, nthread, n_estimators, max_depth, min_child_weight, colsample_bytree, colsample_bylevel, scoring	0.5
Cox Proportional hazard model (CoxPH)		10 folds	Penalizer, random_state	
Gradient Boosting Survival (GBS)		10 folds	n_estimators, learning_rate, max_depth, subsample, random_state	
Multilayer Perceptron (MLP)		10 folds	fc1, fc2, fc_event, fc_time	
Random Survival Forest Survival (RSF)		10 folds	n_estimators, min_samples_split, random_state	

## Data Availability

Data utilized and/or analyzed in this study can be obtained from the corresponding author upon reasonable request.

## Abbreviations

The following abbreviations are used in this manuscript:

AHHREC	Alfred Hospital Human Research Ethics Committee
ANCR	Australian National Cardiac Registry
BARC	Bleeding Academic Research Consortium
CABG	Coronary artery bypass grafting
CAD	Coronary artery disease
CoxPH	Cox proportional hazard
eGFR	estimated Glomerular Filtration Rate
GB	Gradient Booster
GBS	Gradient Boosting Survival
LR	Logistic Regression
LVEF	Left ventricular ejection fraction
ML	Machine learning
MLP	Multilayer perceptron
MI	Myocardial infarction
MICE	Multiple Imputations by Chained Equations
MUHREC	Monash University Human Research Ethics Committee
NCR	National Cardiac Registry
NDI	National Death Index
NHMRC	National Health and Medical Research Council
PCI	Percutaneous coronary intervention
RF	Random forest
RSF	Random Survival Forest
ROC	Receiver operating characteristics
TRIPOD	Transparent Reporting of a multivariable prediction model for Individual Prognosis Or Diagnosis
VCOR	Victorian Cardiac Outcomes Registry
VIF	Variance Inflation Factor
XGB	Extreme gradient booster
